# Characterization and quantification of dynamic left atrioventricular valve regurgitation after atrioventricular septal defect correction with 4D Flow MRI and retrospective valve tracking

**DOI:** 10.1186/1532-429X-16-S1-P138

**Published:** 2014-01-16

**Authors:** Emmeline Calkoen, Jos J Westenberg, Lucia J Kroft, Pieter J van den Boogaard, Monique R Jongbloed, Rob J van der Geest, Albert de Roos, Arno Roest

**Affiliations:** 1 Pediatric cardiology, LUMC, Leiden, Netherlands; 2 Radiology, LUMC, Leiden, Netherlands; 3 Cardiology, LUMC, Leiden, Netherlands

## Background

Regurgitation of the left atrio-ventricular valve (LAVV) is common after atrioventricular septal defect (AVSD) correction and up to 15% of the patients require surgery of the LAVV during follow-up. Reliable quantification of LAVV regurgitation after AVSD correction is essential for indicating surgical correction, but has shown to be difficult using echocardiography. 4DFlow MRI with retrospective valve tracking allows visualization and quantification of trans-valvular blood flow. The aim of cuurent study was to describe dynamic behaviour of regurgitant jets of the LAVV after AVSD correction and to quantify severity of regurgitation using 4DFlow MRI with retrospective valve tracking.

## Methods

**Figure 1:**
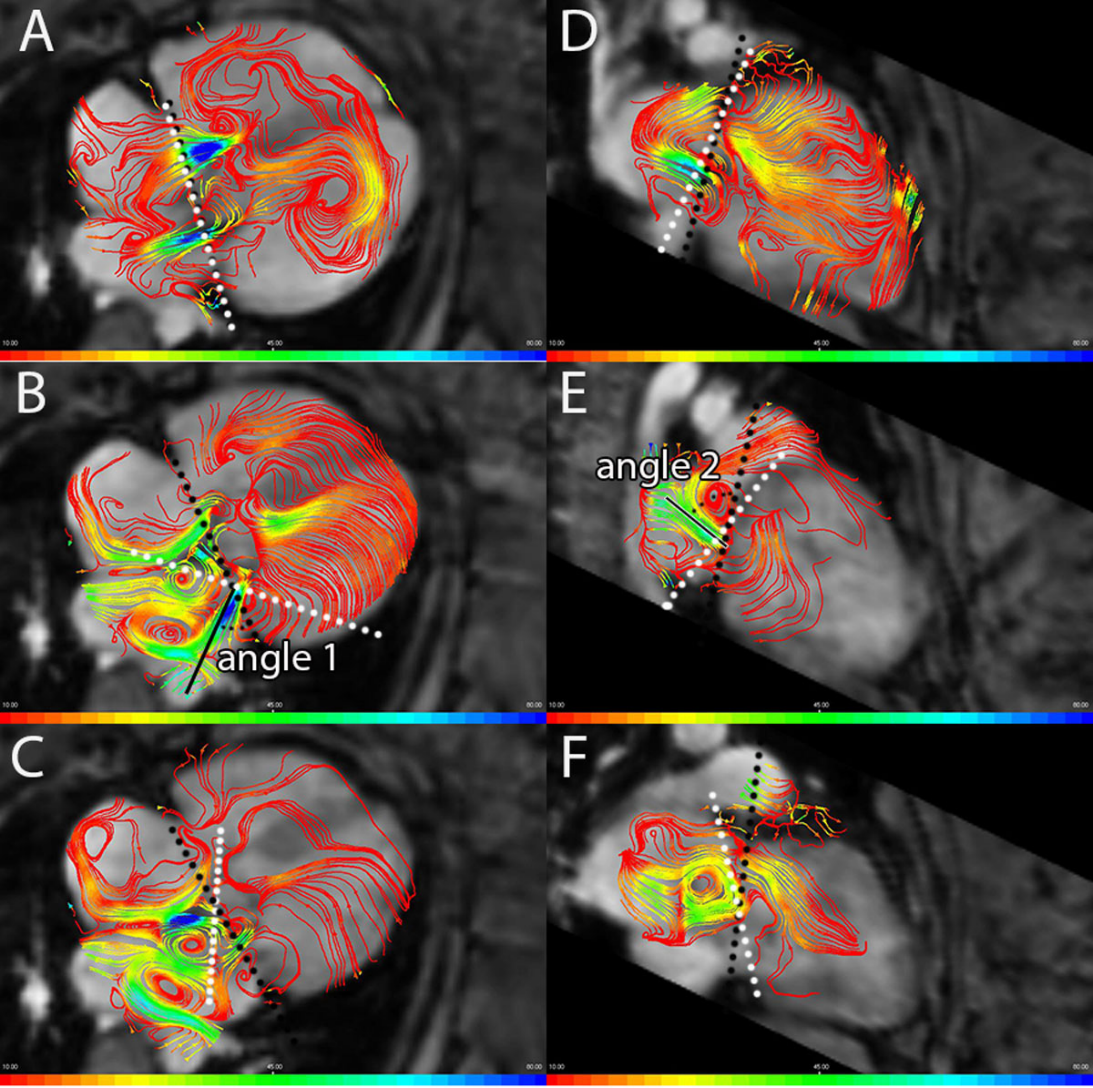
**Multiple dynamic eccentric regurgitation jets in a patient in a 4 chamber (A-C) and 2 chamber (D-F) MPR during early (34 ms) (A, D), mid- (201 ms) (B, E) and late (302 ms)(C, F) systole (total RR 984 ms)**. The jet, visualized with a high velocity by blue streamlines, starts nearly perpendicular (90°) to the annulus (in A and D), but changes to a more lateral and anterior direction (in B and D). A second jet was observed in late systole (in C and F). Black dots show the annulus plane and the white dots represent the orientation of the plane perpendicular to the jet in which regurgitation is quantified (quantification is performed 1-2 cm more proximal to this plane). In B and E, angle measurements between annulus and regurgitation jet are illustrated.

25 patients with a history of corrected AVSD (mean age 23 ± 10 years) underwent whole-heart 4DFlow MRI evaluation on 3T MRI (Ingenia, Philips Medical Systems) with free breathing, velocity encoding of 150 cm/s in all three directions, spatial resolution 2.3 × 2.3 × 3.0 mm 3 and 30 phases reconstructed over one cardiac cycle. Using streamlines, the regurgitant jet was visualized in two orthogonal stacks of parallel cine multiplanar reformatting planes (MPRs) in 2- and 4-chamber orientation, constructed from the magnitude gradient-echo images. At each phase during systole, the MPR with best depiction of the regurgitant jet was used to measure the angle between the jet and the valve annulus (Figure 1). Trans-LAVV and trans-aortic flow were determined from retrospective valve tracking and velocity mapping. Reformat planes during systole were aligned perpendicular to the regurgitation jet visualized with streamlines (Figure 1). Regurgitation velocity was measured 1-2 cm inside the atrium to avoid sampling in an area with phase dispersion.

## Results

17 out of the 25 patients presented with LAVV regurgitant fraction more than 5%. In 5 out of these 17 patients, multiple regurgitant jets were identified. In all patients the angle between the jets and the annulus changed during different phases of systole (Table 1 and Figure 1). Quantification of the regurgitation (regurgitation fraction 16 ± 8%) was feasible with good correlation (R 0.988 p < 0.001) and agreement (mean difference 0.0 ± 3.4 mL; p = 1.0) between LAVV effective forward flow and aorta flow.

**Table 1:** angle between annulus and regurgitation jet

	Four chamber MPR (angle 1)	Two chamber MPR (angle 2)
Maximum angle (degrees)	89 (32)	108 (37)

Minimum angle (degrees)	56 (27)	59 (23)

Angle difference (degrees)	32 (21)	50 (29)

Mean (standard deviation) of maximum and minimum angle between annulus and regurgitation jet throughout systole.

## Conclusions

With 4DFlow MRI and streamline visualization, characterization and quantification of regurgitant jets of the LAVV in patients after AVSD correction is feasible and our findings have important implications for assessment of LAVV regurgitation with echocardiography. Regurgitant jet(s) change dynamically during systole with changes in angle up to 50°, making quantification of LAVV regurgitation using echocardiography or conventional 2D-MRI difficult.

## Funding

Willem Alexander Kinder Fonds and Dutch Technology Foundation (STW) project number 11626.

